# Expansion of the Protein Repertoire in Newly Explored Environments: Human Gut Microbiome Specific Protein Families

**DOI:** 10.1371/journal.pcbi.1000798

**Published:** 2010-06-03

**Authors:** Kyle Ellrott, Lukasz Jaroszewski, Weizhong Li, John C. Wooley, Adam Godzik

**Affiliations:** 1 Joint Center for Structural Genomics, Bioinformatics Core, University of California San Diego, La Jolla, California, United States of America; 2 Joint Center for Structural Genomics, Bioinformatics Core, Burnham Institute for Medical Research, La Jolla, California, United States of America; 3 California Institute for Telecommunications and Information Technology, University of California San Diego, La Jolla, California, United States of America; 4 Joint Center for Molecular Modeling, Burnham Institute for Medical Research, La Jolla, California, United States of America; University College London, United Kingdom

## Abstract

The microbes that inhabit particular environments must be able to perform molecular functions that provide them with a competitive advantage to thrive in those environments. As most molecular functions are performed by proteins and are conserved between related proteins, we can expect that organisms successful in a given environmental niche would contain protein families that are specific for functions that are important in that environment. For instance, the human gut is rich in polysaccharides from the diet or secreted by the host, and is dominated by *Bacteroides*, whose genomes contain highly expanded repertoire of protein families involved in carbohydrate metabolism. To identify other protein families that are specific to this environment, we investigated the distribution of protein families in the currently available human gut genomic and metagenomic data. Using an automated procedure, we identified a group of protein families strongly overrepresented in the human gut. These not only include many families described previously but also, interestingly, a large group of previously unrecognized protein families, which suggests that we still have much to discover about this environment. The identification and analysis of these families could provide us with new information about an environment critical to our health and well being.

## Author Summary

Metagenomics provides a unique opportunity to sample the gene content of microbial communities adapted to specific environments and for the study of the correlations between the presence or absence of gene families that occur in organisms within that environment. Such studies provide detailed information about the adaptation of microbes to a given environment and, indirectly, provide clues about the most important molecular processes that are specific for that environment. Having performed such an analysis for the community of the human distal gut, we report many new protein families and identify many others that are highly specific for this particular environment. The function of most of these proteins is unknown, which illustrates the extent of our ignorance about the organisms within this environment that are so important for human health and well being.

## Introduction

Every ecological niche presents specific challenges that face the population of organisms that inhabit them. When analyzing species that thrive in any particular environment, we can expect that certain key functional characteristics would correlate with success and differentiate those species from others that fail in colonizing that environment. This is especially obvious for microbes, and detailed analysis of almost every sequenced microbial genome provides examples of adaptation, mostly in terms of the presence of genes that code for specific functions required for that microbe to succeed in a given environment. However, studying microbes one genome at a time does not generally provide enough data and meaningful statistics to explore fully the relationships between individual gene families and their environments. This has now changed with the advent of metagenomics, which can investigate entire microbial communities associated with single environments. In metagenomics shotgun sequencing, which identifies genes present in a given environment, the associations between gene families and specific environments can be analyzed directly. All such studies carried out so far have identified unique distributions of functional classes of protein families that are strongly correlated with the specific features of the given environment, be it presence of specific nutrients, acidity, high temperature, etc. For instance, Gill *et al.* have shown that the human gut microbiome is enriched in proteins associated with amino acid and vitamin production [1]. Another study has confirmed these observations and found additional functional groups of proteins overrepresented in the human gut, such as for carbohydrate and lipid transport and metabolism [2]. Similar observations have been made during analysis of the genomes of several human gut–associated microbes, such as *Bacteroides fragilis* [3] and *Bacteriodes thetaiotaomicron* [4]. However, these analyses have focused exclusively on already recognized and functionally characterized protein families—all of which were previously identified and characterized by resources such as PFAM [5], COG [6] or Interpro [7]. As a result, two important groups of protein families were not included in such analyses; namely, families already discovered but not yet characterized, and novel families specific to a newly studied environment but rarely or never found in microbes or in the environments previously studied.. Both sets represent a possible wealth of information about the processes necessary for microbes to survive in the human gut. Their importance for further study was exemplified by a recent metaproteomics study [8], in which almost 20% of all recognized proteins, including several of the most abundant ones, were classified as “hypothetical proteins” and did not belong to well-characterized protein families. Thousands of such environment-specific protein families have also been identified in other environments, such as the ocean [9], [10]. In this study, we address this important issue by an *ab initio* search for protein families in datasets that represent the environment we are studying, and a subsequent abundance/conservation analysis of all protein families, including new examples and those not covered by any functional category.

An important issue in interpreting results of such large-scale studies involves widespread inconsistencies in use of the term “protein family”. While the general definition of a protein family as a group of proteins that evolved from a common ancestor seems very clear, in practical applications, this term can mean anything from a group of very close homologs to an extensive, very divergent group of proteins that shared a common ancestor billions of years ago, but have now evolved into a multitude of sub-families with different functions. Automated procedures for indentifying protein families typically indentify closely related families composed of highly similar proteins, which, upon further analysis, could be included in an already known family or combined with others to form a larger family. Therefore, estimates of the numbers of new protein families provided in large-scale automated project are typically too high. In the context of this paper, we address this problem with detailed analysis of some of the families found in the automated analysis.

The human gut is a very specific environment, rich in diverse nutrients, but also full of challenges for its microbial inhabitants. Because of its richness, the microbes inhabiting human gut form one of the densest microbial communities on Earth, reaching 10^11^ cells per gram [11]. Species that inhabit that environment have to be able to extract energy from diverse and rapidly changing sources, reflecting the diverse human diet that can vary significantly in content and quantity over time in both daily and seasonal cycles. Species forming the human gut microbiome also need to survive encounters with the human immune system and to coexist with other microbes. Sets of specific microbial proteins must carry out the essential tasks of recognizing new nutrients, transporting them into the cytosol and metabolizing them, neutralizing or suppressing human immunity, and signaling to other bacteria and host cells. The presence of genes coding for such proteins in a genome would provide a distinct competitive advantage to a human gut symbiont or commensal microbe.

In this paper, we seek to identify such environmentally specific protein families, focusing on the human gut as a target environment. Because of the obvious importance of this environment for human health, several groups have performed large-scale, random, shotgun sequencing experiments on representative samples providing a direct view of the gene content of this environment [1]–[2]. At the same time, a major sequencing effort, the NIH Human Microbiome Project (HMP), is specifically targeting genomes of human gut microbes [12] as identified, for instance, by 16S rRNA studies. Genomic sequencing provides information for individual species but, with a coordinated effort to sequence the genomes of hundreds of microbes from a single environment, the resultant data can also be translated into an overall gene content. Thus, two sets of independent data can be obtained that describe the gene content of the same environment. Both approaches have their advantages and shortcomings: metagenomic shotgun sequencing provides a relatively unbiased, but small sample of genes that can be found in a given environment. On the other hand, genomic sequencing provides a full set of proteins from a genome, but its success depends on our ability to culture specific species and, thus, might leave large groups of microbes without any representation. Arguably, both of these approaches provide only a very crude approximation of the actual gene content of an environment. However, as we will show, data from both methods present a surprisingly coherent view of the gene content of the human gut, at least on the level of protein families, which encourages us that the data are robust enough for a survey analysis, such as presented here.

We hypothesize that genes coding for proteins that are necessary and beneficial for survival of microbes in the human gut environment will be found abundantly both in the genomes of the species found in that environment and in metagenomic data sampling of the same environment. Hence, we can verify observations made on one set of data by using the other as a reference. At the same time, since an extensive study of the human gut environment and its microbiome was only started very recently, protein family databases and annotation resources, which typically work with significant time lag in recognizing novel protein families, simply haven't had enough time to include data for new families found only in this environment.

In this manuscript, by automated clustering in metagenomics samples from the human gut we identify about 1,800 novel protein families and curate and analyze in detail about 180 of them. Some of these families have been confirmed and characterized by structural studies, since the PSI large-scale Structural Genomics Centers have used a preliminary version of our analysis to select some of the most abundant protein families in the human gut as targets for structural determination [13]. We also present a comprehensive analysis of the distribution of protein families in the human gut environment, including both those previously known, as well as the new families identified in this study.

## Results

In order to identify protein families specific for a given environment, we analyzed random shotgun metagenomic datasets from the selected environment; namely, the human distal gut. Then, we used two reference genome sets to validate and analyze distributions of protein families in the target environment, including both families previously known and the new ones identified in this study. The first set of genomes represents microbes associated with the human distal gut, the same environment as the metagenomics set. As a control, we used another reference set composed of genomes of microbes that have never found in that environment, at least in significant (detectable) quantities. We refer to two these groups of genomes as the Human Gut-Related (HGR) and Human Gut-Unrelated (HGU) sets, respectively. The details of how these two sets of genomes were defined are outlined in the Methods section, and the list of genomes in both groups is provided as a part of Supplemental Materials.

### Novel protein families

**Figure 1:**
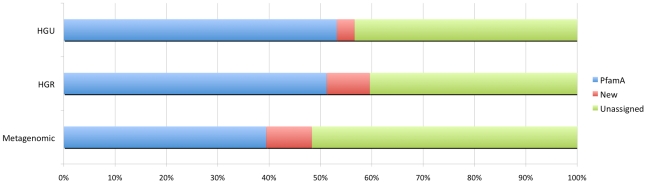
Coverage of genomic and metagenomic datasets with protein families. Sequence sets include Human Gut Related(A), Human Gut Unrelated(B) and Metagenomic sequences(C). The unassigned proteins (green) consist of singletons and small sequence clusters (see text for details).

While many of the ORFs identified in metagenomics shotgun sequencing projects can be classified into already known and defined protein families, many—often over 50% (see Figure 1) —cannot. About 6% are singletons (sometimes called ORFans) [14], i.e., proteins that don't have any homologs in current protein databases. Nevertheless, most of the unclassified proteins do form families of varying sizes and such new families may play very important roles in specific environments, but, by default, were omitted from all previous analyses. In our study, we aim to get a complete picture of protein family distributions in the new environment. To this end, we optimized a previously introduced [10] clustering technique (see the Methods section for details) and used it on the set of over 600,000 ORFs from two large human gut metagenomics projects [1], [2]. We identified almost 1,800 protein families fulfilling our size criteria, of which 926 could be matched to PfamB, the uncurated section of the PFAM database, while the other 835 were found *de novo* in the metagenomic data. We now describe results of various types of analyses applied to these data, including manual curation and experimental verification.

**Figure 2:**
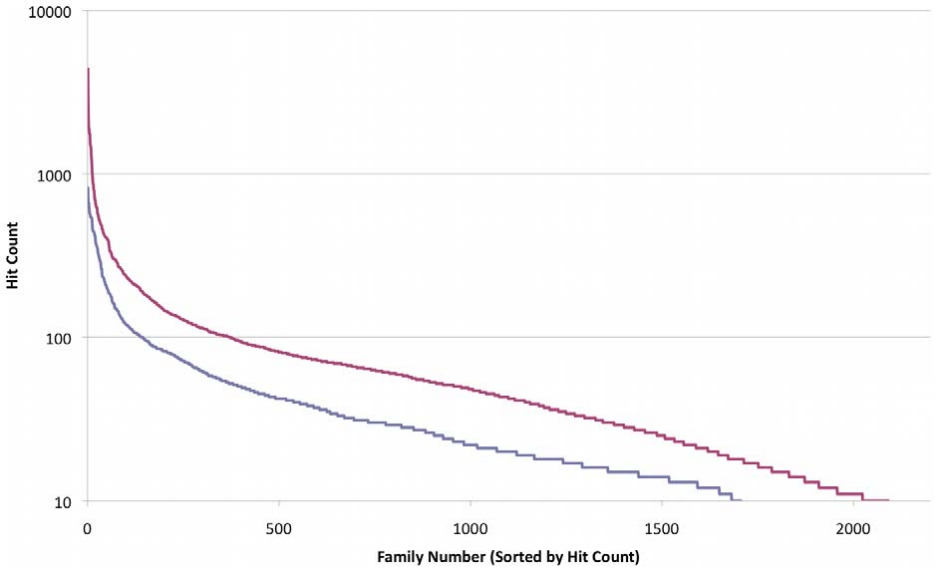
Size distribution of protein families in human gut metagenomics data, PfamA protein families (red) and new families found in this work (blue).

In Figure 2, we compare the distribution of sizes of the new protein families identified here to that of PfamA families that were represented in the metagenomics samples, as sorted by the approximate number of members present in the metagenomic dataset. Both sets have similar size distributions, with PFAM families being somewhat larger. It is interesting to note that only about 2,300 (from over 10,000) PFAM families pass the size threshold (i.e. have ten or more members in the gut-related genomes and metagenomic samples) to be included in this histogram.

### Coverage of human gut genomes and metagenomes

In the next step, we study coverage of the metagenomics datasets, as well as both reference genome sets (HGR and HGU) by the expanded set of families that includes characterized domains from the PFAM database (PfamA) [5], as well as the families newly found in this work (see the previous section). The level of coverage of HGR and HGU genomes by PfamA families is 51% and 52%, respectively. However, the level of coverage drops dramatically to 39% for metagenomic samples. Clearly, while both HGR and metagenomics samples represent the same environment, the metagenomic datasets contain a larger portion of previously uncharacterized genes, most likely from genomes of as-yet-uncharacterized species.

Adding new families identified in this work increases coverage of the metagenomic dataset by approximately 8.9% and increases coverage of reference genome sets by 8.4% and 3.5% for HGR and HGU genomes, respectively. However, in all sets, a large percentage, 40–45% of all ORFs, still cannot be assigned to either an already known or a new family. This group of ORFs can be broadly divided into two groups: a majority (∼88% of the unclassified proteins, i.e. 45% of the total) are proteins that form small families (<10 members), which were not included in the analysis because of the size thresholds used in this work. These “microfamilies” may be an important source of information, but the computational complexity of applying detailed analysis to each of these possible families must await future research. It is very likely that these microfamilies will expand to full-sized families with the addition of new metagenomics datasets, or will be found to be included in already defined families as the sensitivity of their profile description improves with addition of further homologs. The remaining 12% of the unclassified proteins, i.e. 6% of the total, have no BLAST matches internal to the human gut metagenomics samples and, thus, cannot be grouped into clusters of metagenomic sequences. Truly unique protein sequences may be specific to uncharacterized, rare organisms, but it is also possible that they represent failures of sequencing technologies, bad ORF calls, etc. The validity of ORF calls can be monitored; in the analysis of the GOS metagenomics samples, the number of similar sequences has been shown to be strongly correlated with the validity of an ORF call [9], other criteria can be used as well [10].

### Patterns of distribution of protein families in genomes

**Figure 3:**
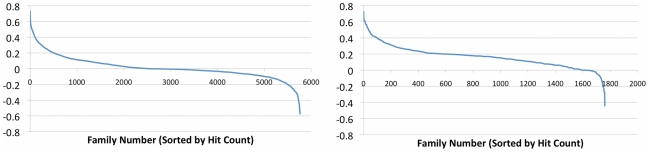
The distribution of “essentiality coefficients” for protein families. PFAM families [5] are shown on the left and the new families introduced in this manuscript on the right panel.

Once a complete set of protein families is identified, the next step is to determine the extent to which these families are specific to our target environment (the human distal gut). To this end, we calculate an “essentiality coefficient” (Es) for every family (see Methods section for a formal definition of Es, as well as for definitions of other measures of environmental specificity of protein families). An essentiality coefficient equal to 1 means that at least one member of a given family was found in the genome of each of the human gut–associated microbes, but no members were found in any of the reference set of genomes—thus, this family is considered as essential for the gut environment. An Es close to 0 indicates lack of preference, and an Es close to −1 indicates an “anti-preference.” Figure 3 presents the distribution of essentiality coefficients for protein families from the PfamA database and for new protein families found in this work.

PfamA protein families show an almost symmetric distribution of preference and anti-preference for genomes of human gut–related microbes. At the same time, the new families found in this work are very specific for human gut–related microbes. This outcome is, of course, expected as these families were identified by clustering from the metagenomics datasets with the aim of identifying environment-specific families. Interestingly, some families that were not specific for the human gut environment were notably found by clustering metagenomics ORFs (lower-right region of graphs in Figure 3b). These are the protein families, found by clustering the metagenomic datasets that turn out to be more frequently conserved in random genomes not connected to that environment. One example is the family HGC00614, composed of 18 proteins found in the metagenomic data. Upon constructing an appropriate HMM, we found that this family is a likely new family in the PFAM PLP_aminotran (CL0061) clan, with many homologs across multiple species. It is also worth noting, that some families found in metagenomic data have not been found in any fully sequenced genomes of microbes from the same environment, clearly showing that complete genome sequencing still hasn't fully explored the diversity of genes present in this environment.

Several different measures can be proposed to compare distributions of a protein family between two datasets. For instance, the comparative overrepresentation (Ov) in a specific dataset details the number of members a family has in one dataset as compared to another reference set. Another metric is the expansion (Ex) of a protein family when the relative counts of protein families are compared but, rather than normalizing by the total number of proteins in the genomic set, counts are normalized by the number of genomes that contain at least one match. This metric highlights families that may not have the largest counts, but when found, have multiple copies in the same genome. Yet another measure is the essentiality coefficient (Es) used above in Figure 3, which compares the percentage of genomes in each group that contain at least one member of a family. So far, we have only used the latter specificity measure (Es). In the following analysis, we will use and compare all three measures as each captures some of the intuitive notion of specificity. Each measure corresponds to a different biological mechanism of “specificity”. Having multiple paralogs of proteins from families with high Es, but low Ov or Ex, clearly does not provide an advantage to a microbe, therefore protein families that score well with Es likely perform highly specific, but essential functions. On the other hand, large number of members of overrepresented or expanded families provides such advantage, but may represent only one of many possibilities of solving a given problem; hence, they are not present in all microbes in a given environment. For instance, metabolic enzymes would likely belong to the latter category, while defense and host signaling proteins would likely belong to the former.

### Human gut microbiome–specific PfamA families

As discussed extensively in the papers that study the human gut microbiome directly through metagenomics sequencing [1], [2] or indirectly through genome sequencing of specific microbes representative of this environment [3], [4], certain protein families involved in specific types of function were observed to be strongly expanded in the human gut microbiome compared to families found in “average” microbes. However, these studies did not cover protein families of unknown function, and focused only on one measure of specificity that is related to our overrepresentation measure Ov, in Table 1. In our analyses, we use and compare three different specificity measures: Ov in Table 1, Ex in Table 2, and Es in Table 3. Our research also focused on complete family coverage, including families of known and unknown function, as well as new families specific for the gut environment. Novel families were ranked by the three different ranking methods, with the top 10 hits listed; Ov in Table 4, Ex in Table 5, and Es in Table 6. The full list of 180 annotated protein families is detailed in Table S1, in the supplemental material.

**Table 1:** The 10 most *overrepresented* (Ov) PfamA families in human gut microbiome.

Family Id	Family name		G	n	G	N	Ov/Ex/Es	PSI
**PF08522**	Domain of unknown function (DUF1735)	Conserved genomic neighbor of SusC/SusD, remote homology to SusE	93	0	12	0	590.68/7.15/0.18	393045, JCSG, Diffraction-quality Crystals
**PF07338**	Osmosensory transporter coiled coil		90	0	8	0	571.62/10.00/0.12	2nocA, NESG
**PF08481**	Protein of unknown function (DUF1202)	Structural protein, probably involved in maintaining cell shape	61	0	6	0	387.43/8.71/0.09	N/A
**PF08800**	VirE N-terminal domain		72	1	12	1	228.65/5.04/0.18	388157, JCSG, Expressed
**PF06603**	GBS Bsp-like repeat		33	0	6	0	209.59/4.71/0.09	APC88089.1, Purified
**PF02920**	DNA binding domain of tn916 integrase		33	0	16	0	209.59/1.94/0.25	N/A
**PF01848**	Protein of unknown function (DUF2534)	Sensory, regulatory proteins	29	0	7	0	184.19/3.63/0.11	Z1342_ECO57, Montreal-Kingston, Expressed
**PF06820**	Enterobacterial EspB protein		25	0	1	0	158.78/12.50/0.02	N/A
**PF04971**	Protein of unknown function (DUF1158)	Cytotoxic phage protein	20	0	7	0	127.03/2.50/0.11	ESSD_ECOLI,, Montreal-Kingston, Cloned
**PF09035**	Excisionase from transposon Tn916		39	1	16	1	123.85/1.79/0.24	N/A

Exact definitions of the Ov category is given in the Methods section. Columns provide numerical values for: g (total number of representatives in genomes of human gut microbiome microbes), n (total number of representatives in genomes of microbes not associated with human gut microbiome), G (number of microbes from human gut microbiome with at least one representative of a family), and N (number of microbes not associated with human gut microbiome with at least one representative of a family). Complete statistics for all Pfam protein families analyzed in this study are provided in the Supplementary Material.

**Table 2 pcbi-1000798-t002:** The 10 most *expanded* (Ex) PfamA families in human gut microbiome.

Family Id	Family name	G	n	G	N	Ov/Ex/Es	PSI
**PF07980**	RagB, SusD and hypothetical proteins	784	54	13	6	90.54/48.29/0.19	3cghA, JCSG
**PF00593**	TonB dependent receptor, branch of the SusC megafamily	1,285	2679	23	171	3.05/37.97/0.01	APC6611, MCSG, Purified
**PF07715**	TonB-dependent Receptor Plug Domain, SusC domain and a branch of the SusC megafamily	1,292	2686	24	172	3.05/36.15/0.02	APC62280.2, MCSG, Crystallized
**PF00419**	Fimbrial protein	205	15	8	8	81.38/21.11/0.11	APC6678, MCSG, Purified
**PF04122**	Spore coat protein (Spore_GerQ)	102	281	2	20	2.30/20.62/−0.01	NYSGXRC-10075, SGX, Soluble
**PF00005**	ABC transporter	4,938	27601	65	493	1.14/18.95/0.00	282417, JCSG, PDB: 1VPL
**PF00165**	Bacterial regulatory helix-turn-helix proteins, AraC family	1,982	3901	62	294	3.23/18.24/0.36	NYSGXRC-11003f, SGX, PDB: 3BT3
**PF01554**	MatE	1,332	1965	63	347	4.30/15.17/0.27	282685, JCSG, Expressed
**PF07495**	Y_Y_Y domain	291	172	17	49	10.68/12.73/0.16	APC81251, MCSG, Cloned
**PF06820**	Enterobacterial EspB protein	25	0	1	0	158.78/12.50/0.02	N/A

Exact definitions of the Ex category is given in the Methods section. For the details of the Table 2 columns see the legend for Table 1.

**Table 3:** The 10 most *essential* (Es) PfamA families in human gut microbiome.

Family Id	Family name	G	n	G	N	Ov/Ex/Es	PSI
**PF02901**	Pyruvate formate lyase	127	151	61	103	5.31/0.60/0.73	NYSGXRC-12027a, SGX, Purified
**PF01228**	Glycine radical	179	219	62	114	5.17/0.94/0.72	NYSGXRC-12027a, SGX, Purified
**PF04204**	Homoserine O-succinyltransferase	58	118	58	114	3.10/−0.04/0.66	2ghrA, JCSG
**PF02659**	Domain of unknown function DUF	131	296	54	92	2.80/−0.80/0.64	N/A
**PF02836**	Glycosyl hydrolases family 2, TIM barrel domain	293	159	52	80	11.63/3.57/0.64	NYSGXRC-12014c, SGX, PDB: 3BGA
**PF01183**	Glycosyl hydrolases family 25	103	106	49	62	6.11/0.38/0.63	388675, JCSG, Crystallized
**PF06738**	Protein of unknown function (DUF1212)	90	203	59	139	2.80/0.05/0.63	APC20809.1, MCSG, Expressed
**PF02837**	Glycosyl hydrolases family 2, sugar binding domain	397	218	52	92	11.51/5.15/0.61	NYSGXRC-12014c, SGX, PDB: 3BGA
**PF00703**	Glycosyl hydrolases family 2, immunoglobulin-like beta-sandwich domain	266	126	48	69	13.30/3.63/0.60	NYSGXRC-12014c, SGX, PDB: 3BGA
**PF10509**	Galactokinase galactose-binding signature	58	150	56	135	2.44/−0.09/0.59	N/A

Exact definitions of the Es category is given in the Methods section. For the details of the Table 3 columns see the legend for Table 1.

**Table 4:** 10 top most *overrepresented* (Ov) new families, from the set of over 180 curated novel families identified in this work.

Family ID	Family description	g	n	G	N	Ov/Ex/Es	Most advanced PSI target (id, center, status)
**PB004588**	No hypothesis about function	105	0	14	0	666.89/7.00/0.22	390317, JCSG, Diffraction-quality Crystals
**PB064361**	Contains putative lipoproteins	60	0	13	0	381.08/4.29/0.20	#N/A
**PB012771**	No hypothesis about function	92	1	21	1	292.16/3.68/0.32	NYSGXRC-T1444, NYSGXRC, Work Stopped
**PB008694**	Contains conserved hypothetical proteins found in conjugate transposon TraH.	40	0	13	0	254.05/2.86/0.20	390153, JCSG, Diffraction-quality crystals
**HGC00311**	No hypothesis about function	35	0	16	0	222.30/2.06/0.25	#N/A
**PB023339**	No hypothesis about function	32	0	13	0	203.24/2.29/0.20	NYSGXRC-12097b, NYSGXRC, Native diffraction data
**HGC00150**	No hypothesis about function	31	0	15	0	196.89/1.94/0.23	#N/A
**PB029229**	No hypothesis about function	28	0	13	0	177.84/2.00/0.20	393207, JCSG, Crystallized
**PB048420**	No hypothesis about function	27	0	18	0	171.49/1.42/0.28	#N/A
**PB047024**	Remote homology to HD domain (PF01966)	24	0	22	0	152.43/1.04/0.34	#N/A

Exact definitions of the Ov category is given in the Methods section. Columns provide numerical values for: g (total number of representatives in genomes of human gut microbiome microbes), n (total number of representatives in genomes of microbes not associated with human gut microbiome), G (number of microbes from human gut microbiome with at least representative of a family) and N (number of microbes not associated with human gut microbiome with at least representative of a family).

**Table 5 pcbi-1000798-t005:** 10 top most *expanded* (Ex) new families, from the set of over 180 curated novel families identified in this work.

Family ID	Family description	g	n	G	N	Ov/Ex/Es	Most advanced PSI target (id, center, status)
**PB155142**	Contains putative TonB-linked outer membrane proteins, part of SusC? Remote homology to several outer membrane receptors	930	158	14	58	37.15/59.32/0.10	#N/A
**HGC00106**	Contains putative TonB-linked outer membrane proteins, part of SusC?	908	76	14	8	74.90/52.09/0.20	APC62223.1, MCSG, Purified
**HGC00024**	N-terminal subdomain of SusD	819	56	14	6	91.26/46.60/0.20	3ejn, JCSG, In PDB
**PB001404**	Branch of SusD family	764	61	13	5	78.27/44.40/0.19	3cgh, JCSG, In PDB
**PB004588**	No hypothesis about function	105	0	14	0	666.89/7.00/0.22	390317, JCSG, Diffraction-quality Crystals
**PB064361**	Contains putative lipoproteins	60	0	13	0	381.08/4.29/0.20	#N/A
**PB012771**	No hypothesis about function	92	1	21	1	292.16/3.68/0.32	NYSGXRC-T1444, NYSGXRC, Work Stopped
**PB000790**	Branch of SusD family	98	17	13	4	34.58/3.60/0.19	3cgh, JCSG, In PDB
**PB202086**	Remote homology to Flagellar basal body-associated protein	21	0	5	0	133.38/3.50/0.08	#N/A

Exact definitions of the Ex category is given in the Methods section. For the details of the Table 5 columns see the legend for Table 4.

**Table 6:** 10 top most *essential* (Es) new families, from the set of over 180 curated novel families identified in this work.

Family ID	Family description	g	n	G	N	Ov/Ex/Es	Most advanced PSI target (id, center, status)
**PB001565**	No hypothesis about function	41	53	41	24	4.82/−1.14/0.58	387995, JCSG, Diffraction-quality crystals
**HGC00044**	Putative glycosyl hydrolase, remote homology to dextranase, polygalacturonase	204	186	44	88	6.93/2.44/0.50	281957, JCSG, Crystallized
**PB004476**	Contains vancomycin b-type resistance proteins vanW, C-terminal domain homologous to L,D-transpeptidase	68	105	37	61	4.07/0.10/0.45	NYSGXRC-10212m, NYSGXRC, Purified
**PB000119**	Contains ABC transporters, remote homology to predicted membrane proteins	129	274	45	129	2.98/0.70/0.43	#N/A
**PB001934**	Small GTP-binding protein	34	45	33	44	4.69/0.00/0.42	389596, JCSG, Diffraction-quality Crystals
**PB019388**	Domain present in radical SAM domain proteins	29	38	29	34	4.72/−0.12/0.38	APC20476, MCSG, Purified
**PB015954**	Contains sortases SrtC	36	3	23	3	57.16/0.75/0.35	#N/A
**PB001030**	No hypothesis about function	27	36	27	36	4.63/−0.01/0.34	APC27927, MCSG, Work Stopped
**PB047024**	Remote homology to HD domain (PF01966)	24	0	22	0	152.43/1.04/0.34	#N/A

Exact definitions of the Es category is given in the Methods section. For the details of the Table 6 columns see the legend for Table 4.

Domains of unknown function (DUF) dominate the overrepresented group with four such families in the top 10 when sorted by overrepresentation (Ov), but the DUFs are also present in other forms of ranking. The presence of so many weakly characterized protein families in all specificity categories clearly illustrates the inadequacy of our knowledge about this important environment. Similarly, all previous analyses focused mostly on metabolic proteins and interpreted the specificity of the human gut environment predominantly in the view of its unique metabolic content. We show here that protein families involved in regulatory and DNA exchange functions are also strongly present among the most overrepresented families.

### Top gut-specific families

It is possible and, indeed, very likely that, by using more sensitive sequence analysis tools, many of the families identified here would be eventually grouped into larger entities, such as clans in PFAM [5] (or superfamilies in other protein classification systems), that represent more distant evolutionary relationships. However, for the purpose of this analysis, we will focus on the family level as practically defined by major community resources, such as PFAM [5] or Interpro [7]. Upon further analysis of the families identified in an automated, *ab initio* clustering of protein sets we realized that many may not fit such definitions. For instance, proteins that form distant branches of already existing families may form well-defined clusters in the automated analysis, but careful optimization of HMMs that define old versus new families would be necessary to decide if they would form a new family or if they could be included in the old family by readjusting its definition. For instance, we found several potential families that belong to the SusC and SusD mega-families. SusC and SusD are part of the sus (starch utilization system) operon in *B. thetaiotamicron*, an archetype of polysaccharide utilization loci found in multiple copies in all *Bacteroides* and related species [15], [16]. Both families are extremely divergent; only a small number of their members are covered by PFAM HMMs that define Ton_B–like and SusD families, respectively. The complex evolution of the SusD protein family is the subject of a separate paper [17].

Families that define new domains in proteins with already recognized PFAM domains form the second group. Again, without detailed analysis, it would be difficult to decide if such families should be defined as new or covered by readjustment of the boundaries of already defined families. We used several filters to identify and remove the group of new families that would be most likely to overlap with already existing PFAM families (see Methods), undoubtedly eliminating some genuine, novel families. Next, we analyzed the remaining ones by hand to identify those that are most likely to conform to the “PFAM standard”, i.e., families that represent functional domains that do not overlap with protein families described in the PFAM database. At this point, the hand-curated set of PFAM-quality families exceeds 180 and would undoubtedly expand further as the curation and analysis continue. We provide the current list of curated families as a Table S3 in the Supplemental Materials.

Tables 4–6 present the top families from this group in three different “specificity” categories. (An analogous table for the Pfam families was presented in the previous section.)

### Experimental verification of novel, human gut–specific protein families

As mentioned earlier, a preliminary version of this analysis was used to select structure determination targets for the four large NIH Protein Structure Initiative production centers in two “target drafts” [13] in mid- and late 2008. As of May 2009, representatives of almost 800 of the 1,761 protein families identified here had been successfully expressed and purified *in vitro*, supporting the conjecture that the new families represent real proteins and not “shadow ORFs” or other sequencing artifacts. The last column in Tables 4–6 provides information about the status of the representative of a given family that is most advanced in the PSI production pipeline.

The structures of representatives of several protein families described here have been successfully solved, and their coordinates deposited into the Protein Data Base. For instance, *Thermotoga maritima* proteins TM1486 (1VPV) and TM841 (1MGP) represent DegV (PF02645), a large family of proteins, shown by structure analysis to be involved in fatty acid binding. The Lactobacillus acidophilus NCFM protein LBA1001, PDB entry 3EDO, incorrectly described in the literature as a TRP repressor, has 142 homologs in metagenomic datasets, and at genomic levels of conservation goes from 12% of species in the HGU sample to 84% in the HGR set. A third example of a protein prevalent in the human gut environment is the protein family represented by PDB entry 2PC1. This acetyltransferase/GNAT family protein has 47 metagenomic homologs and is present in 73% of HGR species, while it is rare in the HGU list (5% of species).

Other protein families determined to be important to the human gut environment and found independently by this study include PfamA family PF08842 (DUF1812), represented by PDB entry 3GF8. Only 3 homologs are found in genomes of free-living bacteria (HGU set), compared to 47 in the of human gut-related microbes. The proteins matching this family in the HGU genomes were found to be hypothetical proteins in *Porphyromonas gingivitis*, an human oral pathogen, which was included in the HGU set because of its specific definition (see Methods), but should probably be reclassified to the HGR set. The Protein Structure Initiative has also solved several proteins from family PB002962 (PDB entries 3DB7 and 3DUE). This family was found in eight of the thirteen of the metagenomic samples, with a total of 34 homologs and present in only 1.6% of HGU genomes as compared to 21.5% of HGR genomes.

## Discussion

The gastrointestinal tract is extremely important for overall human health. Numerous diseases, from digestive disorders and immune diseases to numerous types of cancer, notably involve the GI system. At the same time, the human GI system, and especially, the distal gut, is a surprisingly complex and little understood environment, inhabited by a complicated bacterial community that carry enzymes for processing byproducts and downstream products of metabolism in the stomach and proximal gut. Rich in nutrients, the gut harbors one of the densest microbial populations known. These microbes and their metabolism play a critical role both in health and in diseases of the GI system. While the culturable microbes living in the human gut have been studied for decades (for instance, *E. coli*), the development of new technologies and the concept of metagenomics provided a decisive, paradigm-changing shift in studies of this environment, in which the diversity and the communal nature of the human gut microbiome could be uncovered. We thus now have access to several synergistic, but independent, lines of investigation into the surprisingly unknown world of microbes inhabiting human cavities. Here, we investigated what types and number of novel, previously uncharacterized, protein families can be found in this environment. In our analysis, we have shown that many protein families, most completely uncharacterized, show strong specificity for this environment. Undoubtedly, the functions of these proteins play an important role in the maintenance and operation of the human gut microbiome. Approximate function predictions based on distant homology recognition identified many proteins that are involved not only in metabolism, but also in signaling, regulation, and phage activity, and are obviously very important in such dense bacterial communities.

We have identified not only a few thousand known protein families as strongly overrepresented in the human gut environment, but also, many potentially new protein families. Many of these assignments have now been confirmed by structural determination by the PSI centers, and many of their functions have been predicted due to fold recognition techniques. However, many yet uncharacterized or completely novel families have been shown to be specific to the human gut environment. This observation, in turn, suggests that many unknown and uncharacterized processes are yet to be discovered in this environment.

Apart from these interesting insights about this specific environment, our observations suggest this approach is applicable to analyzing other environments. Historically, genomic analysis has focused on individual species, but it is important to remember that an organism does not exist in a vacuum. Organisms evolved their specific traits in the context of their environment. By sampling the gene pools in a given environment, we can learn about the protein families that are key for survival in those environments. The methods presented here should aid in organizing and streamlining such analyses.

## Methods

### Data preparation

Our analysis is derived from several different sources: metagenomic sequencing, 16S rRNA sampling, fully sequenced cultured genomes from NCBI, and draft genomes published by the Human Gut Microbiome Initiative (HGMI) [12]. Each of these data sources is publicly available.

We used a human gut metagenomic dataset derived from the Kurokawa [2] study. This dataset contains 350,000 assembled contigs from 13 individuals, both male and female, with ages ranging from 3 months to 45 years. Although these genomic data come from 13 separate individuals, we have treated them as a single set to improve the odds of finding human gut–related proteins. Preparation of the sequence metagenomic data begins with Open Reading Frame (ORF) prediction done by Metagene [18]. Metagene analysis produced a set of 665,559 ORFs. From this initial set, incomplete ORFs that ran off the edge of the sequence read were removed. A total of 303,314 complete ORFs were left. This set was then used to identify protein families (see the section *Clustering and identification of uncharacterized and new families*).

The HGMI sequenced genomes provide an ideal reference set of human gut–related microbial genomes. In addition to the human gut–related reference genomes (HGR), we also needed a set of genomes not related to the human gut environment for comparative analysis. The set of selected fully sequenced genomes was derived from the collection of bacterial genomes available from NCBI. As of July 2008, this library included 765 bacterial genomes. We utilized data from 16S rRNA sampling to eliminate genomes linked to the human gut environment by targeted metagenomic sampling. The 16S rRNA data was derived from two sources: Greengenes [19] and David Relmann's published human gut sample 16S RNA set [20]. Using data available in the Green Genes, we searched for 16S rRNA sequences associated with keywords “human” and one of the following: “fecal,” “faecal,” “colon,” “intestine,” “stool,” “rectum,” “cecum,” “feces,” “intestinal,” “colitis,” “stomach,” or “gut.” This search produced a set of 38,839 16S rRNA sequences. This set was added to the 11,831 sequences from the Relman dataset. Using a broad Operational Taxonomic Unit (OTU) of 90% sequence identity, we ran BLAST against the set of NCBI bacterial genomes and selected 493 species not linked to the human gut microbiome (i.e. those which did not match any 16S RNA sequences from species related to human gut). We refer to this latter set as the Human Gut–Unrelated (HGU) set.

To create the set of Human Gut–Related genomes, we started with 45 genomes from the HGMI project, each currently in the draft stage. In addition to that base set, we added 20 finalized NCBI bacterial genomes tagged with matching 16S rRNA sequences that were manually confirmed by examining NCBI genome project annotations. This provided us with a set of 65 genomes referred to as the Human Gut–Related (HGR) set. Detailed information about both sets is available in the Table S2 in Supplemental Materials.

### Clustering and identification of uncharacterized and new families

One of the important aspects of analysis of metagenomic sequences is the identification of novel sequences. These sequences with no known homolog in existing sequence databases are referred to as orphan sequences. In the study by Kurokawa *et al.* [2], orphan analysis was carried out by taking over 600,000 predicted ORFs and looking for genes previously seen with BlastP with a threshold of 1.0e-5 against a custom, extended, non-redundant (NR), sequence database. Of the original set, 162,647 genes were determined to be orphan sequences. This set was combined with 503,115 other orphan genes from other metagenomic environments. The total set of orphans was calculated by producing an all-to-all BlastP [21] comparison. Connections were drawn between proteins with alignments that had a Blast score of 60 or greater and were marked as a match and the connection graph was then clustered with TribeMCL [22].

The main difference between our analysis and that of Kurokawa *et al.* is that they augment their human gut metagenomic ORF orphan set with orphans from other metagenomic environments. We believe the main benefit of metagenomic sequencing is that protein families related to specific environments can be targeted. These environmentally specific signals may have been lost by adding sequences from other environments.

In our study focused on identification of novel and uncharacterized protein families we used the procedure described below (outline of the procedure is also give in a separate table (T1) in the supplement materials).

We used the set of metagenomic sequences prepared as described in the *Data Preparation* section earlier. It includes 303,314 complete ORFs. In the first step of the analysis we removed metagenomic sequences that belong to families annotated in PfamA database. This was done by masking all fragments which were aligned with HMMs representing PfamA families (we used hhmscan from HMMER package [23]). Subsequently we identified uncharacterized and putative novel families in the remaining (i.e. unmasked) sequences.

#### Identification of uncharacterized families (PfamB families)

In the next step of our analysis we focused on potential protein families that have been previously seen, but not yet characterized. We used PfamB [24], which is an automatically generated library of possible sequence domains generated by analysis of the Uniprot protein space, after masking hits to PfamA families in Uniprot. With over 200,000 possible multiple sequence alignments in PfamB, it is currently computationally prohibitive to run all of the PfamB HMMs against the metagenomic data. Therefore, we scanned metagenomic ORFs against the complete set of PfamB sequences using Blast. PfamB families where seed sequence had at least 15 hits in metagenomic set were accepted. As many as 1636 PfamB families passed that criterion and were included in further analysis where their HMMs were used to scan the genomic and metagenomic sets.

#### Identification of novel families

Alignments of PfamB sequences were used to produce HMMs representing PfamB families (using hmmbuild program from HMMER package) and then fragments of metagenomic sequences that matched PfamB HMMs were masked. The remaining fragments were clustered with TribeMCL in a manner similar to that performed in the study by Kurokawa et al. After this initial clustering, each of the representative sequences were used to produce position-specific score matrices using five iterations of Psi-Blast [21], [25], over a sequence database consisting of the NCBI NR database and metagenomic sequences reduced with CD-HIT [26] to clusters of 85% sequence identity (this database is further referred to as NR85s). These profiles were then scanned against the metagenomic samples to look for protein clusters that have overlapping hits. Overlapping clusters were merged, and multiple sequences alignments were created. At this point, HMMs were created for each of the 1,216 putative novel families produced by this procedure.

#### Elimination of uncharacterized and putative new families overlapping with PfamA

The HMMs for uncharacterized families (PfamB domains) and putative new families were created from multiple sequence alignments of sequences collected by running five iterations Psi-Blast searches using the longest representative sequence from a family against the NR85s database. Then, hmmbuild program from HMMER package [23] was used to prepare HMMs from the resulting alignments. Once a family HMM had been produced, the unmasked metagenomic set was rescanned and overlaps with existing PfamA families were checked. Families that had overlapping hits with any particular PfamA family in more than 20% of collected sequences were removed. This left a set of 1,761 protein families (926 uncharacterized PfamB families and 835 novel families found by metagenomic clustering).

#### Counting hits in HGR and HGU genomes

Once the clustering was done, statistics was collected on each of the families based on their HMMs. HMM scans were then carried out against the HGR and HGU genome sets (for definitions see the Data preparation section earlier) with hmmsearch program from the HMMER package [23] using an e-value cutoff of 1e-4 and a database normalization constant of 2,000. Numbers of matches in each of these sets were counted and used to calculate overrepresentation (Ov), expansion (Ex), and essentiality parameters (see the section Analysis of representation of protein families in human gut microbiome below).

### Additional filtering

Our method to produce automatically derived human gut–related uncharacterized sequence clusters yielded 1,761 putative protein families. By analyzing the families, we found several common flaws and characteristics that created less-than-optimal automatic family descriptions. Initially, by taking the target HMMs and rescanning the metagenomic dataset, we found that 112 models produced no hits within the cutoff value. To eliminate bad data or uninteresting results, we set up a series of criteria to filter possible families. One of the most common problems was identification of families that had not been fully detected by the existing PfamA models but, in fact, were branches of annotated PfamA families. After applying FFAS to detect similarities between families found by the clustering technique and PfamA families, we removed families with a good probability of being linked to existing PfamA families. This initial filter reduced the set of families to 1,250. The next step was to remove possible clusters of incomplete proteins. We found many cases of sequence fragment clusters that were, in fact, associated with PfamA families, but not described by the HMM representing a family. In these cases, the PfamA model only describes the most conserved subsection of the family alignment and by clustering, we collected sequence fragments left outside the HMM. To filter these clusters, we accepted only families that could be aligned with at least 75% of at least one protein found in a known genome. This filter reduced the number of families to 486. We also filtered using the length of the HHM representing the proposed family to remove sequences unlikely to represent full protein domains. After removing families represented by HMMs shorter than 100 elements, the number of families dropped to 317. Another problem was the presence of HMMs that produced fewer hits in the metagenomic sequences than the original set of sequences used to create them. By removing clusters with fewer then 10 metagenomic hits, the number of protein families dropped to 291. All 291 candidates for new families were subsequently manually curated by inspecting Psi-Blast and FFAS results and the following cases were removed from the list:

- Families with ‘divergent’ Psi-Blast results i.e. cases where Psi-Blast alignment did not contain any motifs conserved in all family members and/or includes many low complexity sequences.
- Families where Psi-Blast alignment consisted of short fragments aligned with different regions of the query (no apparent family ‘core’)
- Families with less than 15 Psi-Blast hits in nr database
- Families that, despite lack of HMMER hits in Pfam database, can be easily assigned function based on annotations of individual proteins (thus not new nor uncharacterized)

Manual curation eliminated 111 families reducing the number of families to 183. Full list of the curated families is provided in the Table S3 in the Supplemental Materials.

### Analysis of representation of protein families in human gut microbiome

We proposed three parameters to evaluate overrepresentation of protein families in the human gut microbiome: comparative overrepresentation (Ov), expansion (Ex), and essentiality (Es) defined by the following formulae:
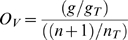


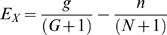


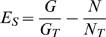
where:

g, n represent the number of occurrences of a family in all genomes from the human gut microbiome and the number of occurrences in genomes not present in the human gut microbiome.
g_T_, n_T_ represent the total number of proteins from the human gut microbiome (included in this analysis) and the total number of proteins from genomes not related to the human gut microbiome (included in this analysis). In our study, g_T_ = 224,099 and n_T_ = 1,423,331.
G, N represent the number of genomes from the human gut microbiome with at least one occurrence of a family and the analogous number for genomes not present in the human gut microbiome.
G_T_, N_T_ represent the total number of genomes from the human gut microbiome (included in this analysis) and the total number of genomes not related to the human gut microbiome (included in this analysis). In our study, G_T_ = 65 and N_T_ = 493.

Numerical values of each of overrepresentation, expansion, and essentiality were calculated for all new families identified by our analysis and also, separately for all families from the PfamA database (see Supplementary Materials). The top-ranking PfamA families and new families are shown in Tables 1–3 and 4–6, respectively.

## Supporting Information

Table S1 Procedure for clustering and identification of uncharacterized and new families.
(0.09 MB DOCX)

Table S2 The list of genomes in the human gut-related (HGR) and human gut-unrelated (HGU) sets.
(0.07 MB XLS)

Table S3 The list of 182 curated new families specific to the human gut environment.
(0.29 MB XLS)
