# Supplementary material for: Expansion of the Protein Repertoire in Newly Explored Environments: Human Gut Microbiome Specific Protein Families
Source: PLoS Comput Biol. 2010 Jun 3;6(6):e1000798. doi: 10.1371/journal.pcbi.1000798 (PMC2880560; doi:10.1371/journal.pcbi.1000798)
Supplement: Table S1 — Procedure for clustering and identification of uncharacterized and new families. (0.09 MB DOCX) [file pcbi.1000798.s001.docx]

**Table S1:** Procedure for clustering and identification of uncharacterized and new families

1) Find PfamA matches in the metagenomic set and mask them

*Identification of uncharacterized families (PfamB families):*

2) Scan remaining (i.e. unmasked) sequences with PfamB seed sequences using Blast

3) Select PfamB families with 15 or more Blast hit and generate HMMs for them

*Identification of novel families:*

4) Rescan metagenomic sequence with generated HMMs of selected PfamB families (see pt. 3)

5) Mask metagenomic sequences aligned with any PfamA or PfamB family

6) Cluster remaining (i.e. unmasked) sequences with TribeMCL using graph based on alignments from Blast

7) Select sequence representatives from clusters and generate position-specific sequence matrices (PSSMs) by performing Psi-Blast searches against NR85s

8) Use PSSMs to collect matches from metagenomic sequences

9) Merge clusters with overlapping sequence hits from searches with PSSMs (see pt. 9)

10) Generate HMMs based on merged clusters and additional hits found in NR85s database with PSSMs

*Further elimination of uncharacterized and putative new families overlapping with PfamA families*

11) Scan the original (not masked) set of metagenomic sequences with HMMs of new and uncharacterized families and remove families where more than 20% of protein matches were also found with HMMs of PfamA families.

*Counting hits in HGR and HGU genomes*

12) HMMs of uncharacterized and new families were used to collect matches in human gut related genomes (HGR set) and in human gut unrelated genomes (HGU set)
